# One change, different effects: the impacts of reducing clerkship length

**DOI:** 10.1186/s12909-021-02732-6

**Published:** 2021-05-21

**Authors:** Blair A. Reece, K. Ramsey McGowen, Kenneth E. Olive, Catherine R. Peeples

**Affiliations:** 1 Department of Internal Medicine, East Tennessee State University Quillen College of Medicine, 325 N State of Franklin, 2nd Floor, Johnson City, TN 37604 USA; 2 Department of Psychiatry and Behavioral Sciences, East Tennessee State University Quillen College of Medicine, Office of Academic Affairs, Box 70571, Johnson City, TN 37614 USA; 3 Department of Internal Medicine, East Tennessee State University Quillen College of Medicine, Office of Academic Affairs, Box 70571, Johnson City, TN 37614 USA; 4 Office of Academic Affairs, East Tennessee State University Quillen College of Medicine, Office of Academic Affairs, Box 70571, Johnson City, TN 37614 USA

**Keywords:** Curriculum change, Clerkship length, Multiple stakeholders, Schools, medical, Data management, Curriculum, Academic performance

## Abstract

**Background:**

Medical school curricula are constantly evolving and change has potential positive and negative effects. At East Tennessee State University Quillen College of Medicine, a broader understanding of the effects of a curriculum change (reduction in clerkship length for one transitional year) was explored.

**Methods:**

A broad, system-wide evaluation was used to evaluate impacts on all stakeholders. Curriculum management data, including qualitative and quantitative data and short-term and follow-up perspectives of stakeholders, were used for evaluation.

**Results:**

Students evaluated the change positively. Academic performance in the transitional year was similar to the prior year. Differences in students’ clerkship evaluations were not statistically significant. Clerkship directors were concerned that students’ clinical experience suffered and noted that implementing changes was time consuming but recognized the benefits for students. Administrators dedicated a significant amount of time to planning the transitional year; however, the additional weeks at the beginning of fourth year made the scheduling process easier.

**Conclusion:**

This article demonstrates an overall positive result with this tool for curriculum change but also indicates the impacts differed across stakeholders. Knowledge gained from this experience can help other schools successfully anticipate challenges and prepare for a variety of outcomes in implementing necessary curriculum change.

## Background

The pace of change in undergraduate medical curricula is rapid and its scope is broad. Understanding outcomes associated with various changes is essential to determine the success of innovations and learn the right lessons from changes [1, 2]. While many studies have reported outcomes related to implemented changes, they often focus on a limited effect such as standardized test performance or limited educational and student outcomes [3–5]. Others present student perceptions, faculty experiences, or leadership perspectives [6–8].

These limited reports provide essential information but do not allow institutions to assess the simultaneous impact of change on all stakeholders. Recommendations for planning curriculum change advise considering the vantage points of all stakeholders, such as students, faculty, and administrators [9, 10]. Coordination of effort between stakeholders is required. It is possible that a change that benefits one can have adverse effects on another.

Our institution implemented a curriculum change that included beginning the fourth-year earlier. Clinical resources did not permit overlapping two classes for the time required to accomplish this change. To assure third-year students completed their clinical rotations before fourth-year students arrived, each third-year clerkship was decreased in duration by 1 week for one transitional year. After this transitional year, the third-year clerkships returned to their traditional durations. We analyzed routinely collected data used for curriculum management to perform a comprehensive evaluation of this third-year change on the whole medical education program. We evaluated the effect of the process used to implement this change as well as the impact on a variety of objective outcomes and subjective experiences of multiple stakeholders. By widening the focus of the evaluation, we provide information about system-wide effects that can assist other schools in anticipating a wide range of consequences associated with curriculum adjustments. The change was planned as a means to improve the clinical curriculum, but we anticipated there would be costs of the change.

## Methods

East Tennessee State University (ETSU) Quillen College of Medicine is a small, community based medical school in Northeast Tennessee. Quillen has two curriculum tracks, a generalist track and an optional rural primary care track (RPCT) that enrolls approximately 20% of the class. To examine the results of a 2018–2019 curriculum change on students, we primarily compared the class of 2020 (transitional year, *n* = 68) to the previous year’s class (standard year, *n* = 72). The two classes were similar in their gender makeup, age at matriculation and entering GPA and MCAT score percentile. Clerkship directors and academic administrators did not change between the years, allowing for comparability.

After conducting a comprehensive curriculum review, we found there was a need for fourth-year students to have additional time for personalized career exploration and away rotations prior to the opening of the Electronic Residency Application Service (ERAS). Additional time would also facilitate students having credentials such as Step 2 scores prior to applying to residency and would ease scheduling of required fourth-year experiences. As a result, in 2018–2019 we modified the third-year academic calendar to enable starting the 2019–2020 fourth-year curriculum 6 weeks earlier. To reset the academic calendar, a one-time shortening of the duration of third year clerkships (the transitional year) occurred because clinical sites could not accommodate twice the number of trainees simultaneously.

Before this change, generalist track third-year clerkships consisted of 8-week rotations in internal medicine and surgery and 6-week rotations in family medicine (FM), obstetrics and gynecology (Ob/Gyn), pediatrics, psychiatry and community medicine. Students in the rural track (RPCT) completed the same clerkship curriculum except for a 12-week clerkship in a rural location that replaces the FM and community medicine clerkships. During the transitional year, all generalist track clerkships were shortened by 1 week (12.5% reduction of 8-week clerkships and 16.7% reduction of 6-week clerkships) and the RPCT clerkship was reduced by 2 weeks (equivalent to the 16.7% reduction on FM and community medicine). The standard length of clerkships was restored after the transitional year. Table 1 represents a sample schedule, requirements, and grading allocation for one clerkship during the transitional year and standard year.

**Table 1:** Sample clerkship schedule comparing the two years

	Standard Year	Transitional Year	Change
Schedule	Total: 8 weeks 6 weeks general internal medicine wards split into 2 sites 2 weeks elective rotations	Total: 7 weeks 6 weeks general internal medicine wards split into 2 sites 1 week elective rotation	Transition year reduced elective time by 1 week
Requirements	6 history and physical exam write-ups for review by an attending 6 overnight calls 6 four hour didactic sessions 5 EKG interpretations 1 mid-clerkship review 1 observed history and physical exam by a supervisor 1 review and interpret peripheral blood smear 1 interpret and report urinalysis results	5 history and physical exam write-ups for review by an attending 6 overnight calls 5 four hour didactic sessions 4 EKG interpretation 1 mid-clerkship review 1 observed history and physical exam by a supervisor 1 review and interpret peripheral blood smear 1 interpret and report urinalysis results	Transitional year reduced number of didactic sessions, required history and physical exam write-ups, and EKG interpretations
Grade components	35% Faculty observations 35% NBME 20% Graded Quizzes 10% Graded H&P	40% Faculty observations 30% NBME 20% Graded Quizzes 10% Graded H&P	Transitional year increased proportion of grade allocated to faculty observation and reduced percentage allocated to NBME subject exam

As part of routine program evaluation, we collected data on objective educational outcomes, such as Step 2 scores and pass rates, NBME subject exam score, and clerkship grades, and subjective experiences of students, faculty, and administrators. In addition to comparing student performance data from the transitional year to prior years, we conducted follow-up surveys on faculty and students after 1 year to evaluate perceptions of the process after the standard schedule was restored and a longer-term perspective could be applied. Further explanation of the surveys are included in the Results section.

The impact on administrators was evaluated though changes in fourth-year scheduling opportunities, their informal impressions, costs, and the number of educational program discussions related to the transitional year made by the curriculum committee. All these data together provide a comprehensive, system-wide evaluation.

## Results

### Educational outcomes

During the transitional year, all students completed all clinical procedures needed to satisfy College of Medicine required procedures. Minor modifications were made to some clerkship requirements to accommodate the shortened schedule, including reducing the number of required patient types to satisfy clerkship objectives. This change was planned by clerkship directors prior to the transitional year and reflected a level of patient encounters that clerkship directors believed satisfied educational objectives.

In five of eight clerkships, the students in the transitional year performed as well or better than those in the standard year, as demonstrated by the percent of students receiving an “A” on the clerkship. In internal medicine and Ob/Gyn, the transitional year students received fewer A’s than those in the standard year, but this difference was not statistically significant. In one clerkship, psychiatry, the proportion of the transitional year class receiving an A was significantly lower than the standard year class (67% vs 84%, χ^2^ = 4.47, *P* = .035).

**Fig. 1:**
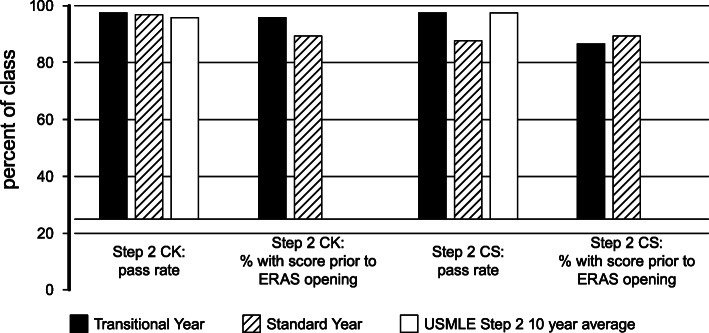
Step exam results

Passing rates on Step 2 CK and CS and the date when Step 2 scores were received were compared between classes and to the 10-year average for our school. Comparison to the 10-year average was conducted because the standard year passing rate on Step 2 CS was significantly lower than the historical passing rates for the school and therefore did not provide an effective comparison for the transitional year. Transitional year students took Step 2 CK earlier, with more students receiving scores before applying to residency (95.65% vs 87.7%, respectively). The transitional year class was not more likely to have a score on Step 2 CS prior to applying to residency. These results are presented in Fig. 1. (Fig. 1).

**Fig. 2:**
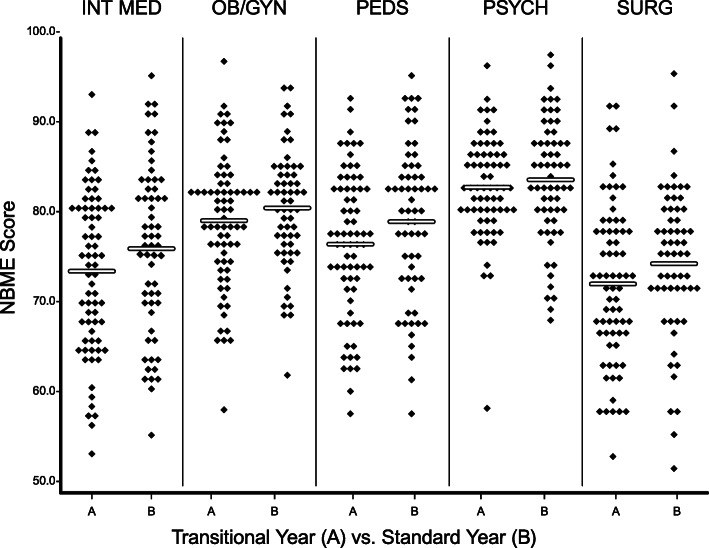
Scatter Plot comparison of NBME subject exam scores

Five clerkships used a NBME subject exam as an end-of-clerkship summative assessment. FM and RPCT used the examination developed by Aquifer, a nonprofit medical education organization. The Aquifer exam is a nationally-normed summative examination based on the Family Medicine clerkship curriculum. It is an alternative product to the NBME subject exam. On the NBME subject exams, all transitional year clerkships had a decrease in the mean score; however, none of the decreases were statistically significant. The top to bottom distribution of grades was similar. (Fig. 2). Performance on the Aquifer exam was significantly lower in the transitional year. The mean score for the transitional year was 79.43 compared to 85.48 for the standard year (t = 4.24, *P* < .01).

### Student and faculty evaluation of clerkships

Students evaluate each clerkship at the end of every clerkship period and provide an overall global rating captured in the response to the question “My overall evaluation of this clerkship is” (5 = excellent; 1 = poor). These overall ratings were averaged for each clerkship period at the end of the academic year, in accordance with acceptable statistical practice [11]. Comparison of transitional and standard year ratings showed minimal difference with no trend in direction. There were no meaningful differences in rural track and generalist track. All differences were statistically non-significant. (Table 2).

**Table 2:** Comparison of student overall ratings of clerkships in transitional and standard years

Clerkship	Transitional Year	Standard
Family medicine	4.73	4.74
Internal Medicine	4.40	4.23
Ob/Gyn	4.53	4.16
Pediatrics	4.44	4.37
Psychiatry	4.30	4.35
Surgery	3.99	4.03
Community Medicine	3.28	3.70
RPCT (20% of class)	4.53	4.58

Students also rated the third-year curriculum after completing the entire year. The Retrospective Survey of the Curriculum is administered to all students at the conclusion of each academic year and asks students to anonymously re-evaluate the components of the completed year. Retrospective surveys were completed by fifty-two transitional year students and 45 standard year students. Ratings on the question “My overall rating of the M3 curriculum is” indicated no change. This global rating for the transitional compared to standard year was, respectively, 3.87 versus 3.71 (5 = very satisfied; 1 = very dissatisfied).

Additional follow-up was obtained through a routinely administered survey of fourth year students immediately prior to graduation. For the transitional year class, the survey included 10 questions about the transitional year. Questions focused on how they used the extra time in the fourth year, their degree of agreement/disagreement with statements about the value of activities, plus a final appraisal of whether the benefits to the fourth year were worth the challenges of the transitional year changes. Ninety-seven percent of respondents believed the benefits offset transitional year challenges. Students identified the top three benefits as increased confidence in specialty choice, the opportunity to take Step 2 CK earlier, and increased confidence in taking Step 2 CK. These results are reported in Table 3.

**Table 3:** Student Ratings of Impact of Transitional Year (*n* = 60)

Item	% Agree/ Strongly Agree
It was the right decision to implement the transitional year	96.61
It provided additional career exploration that increased my confidence in specialty choice	91.66
It allowed me to take Step 2 CK earlier	86.44
It increased my confidence in taking Step 2 CK	86.14
It allowed me to take away rotations that enhanced my chances of matching into a specialty program	84.48
It increased my confidence in taking Step 2 CS	81.03
It allowed me to take Step 2 CS earlier	74.58

Clerkship directors received an 8-item survey 1 year after the end of the transitional year. Four clerkship directors (50%) responded. Clerkship directors offered mixed assessments of the shortened year. Generally, they believed that students benefited from the additional time for career exploration and board examinations. Two rated making the required changes as difficult and two rated it as neither easy nor difficult; none rated it as easy. They were divided over whether shortening clerkship duration was the right approach to use for accomplishing the benefits.

The faculty measure was the clerkship director self-study which clerkship directors complete at the mid-point for each academic year. They report educational outcomes, evaluate clerkship strengths and weaknesses, and provide information to the curriculum committee about issues and concerns. Clerkship directors provide narrative responses on most items. The comments offered about the impact of the transitional year were reviewed and synthesized. These were distilled into negative, positive and neutral effects, with each category being roughly equal. These are summarized in Table 4.

**Table 4:** Themes of Clerkship Director perceptions of transitional year with shortened clerkship lengths (*n* = 8)

Negative Effects	Positive Effects	Neutral Effects
Educational experience less cohesive	Accelerated adoption of changes that addressed identified problems	Changed assessment methods in anticipation of changed clinical experiences
Overall quality of clinical experience diminished	Adoption of new assessment methods that benefitted students (e.g., weekly quizzes)	Changes in weighting of grading components
Preceptors less familiar with students because of reduced time	Development of new clinical resources and placements	Time limited nature of transitional year provided reassurance that change was manageable
Accomplishing educational objectives more difficult	Calendar change facilitated scheduling of introduction to clerkships experiences because of increased availability (occurred in May instead of late June)	
Implementing changes was time consuming		
Shortened time exacerbated pre-existing challenges		

### Administrative outcomes

After controlling for class size, the transitional year class completed 91 more fourth-year clinical experiences than the standard year class prior to ERAS opening. In evaluating the scheduling process, the medical education director for clinical years indicated the additional weeks eased the process significantly. There was no direct financial cost to the medical school; however, there was an indirect time expense for many faculty members and administrators alike. The curriculum committee devoted significant time to planning and addressing issues related to the transitional year: about one-quarter of meetings included agenda items related to the transitional year in the years adjacent to and including the transitional year (9 of 35 meetings). Some of these included policy issues such as revising how clerkships employed NBME subject exams to determine grades or passing criteria.

## Discussion

The medical school curriculum is ever-evolving. While change is intended to be positive and propel educational improvement, it can be logistically challenging and outcomes are not known in advance. We believed that the shift in the academic calendar and starting the fourth-year earlier would be positive, but we did not know if the challenges posed by the required transitional year and shortened clerkships would be worth the benefits. With this system-wide evaluation, we demonstrated an overall positive result but also showed that the impact differed across stakeholders.

Students accomplished more career exploration, had valuable experiences to enhance successful residency acceptance, and had improved rates of credentials in hand prior to ERAS opening. Students in transitional year clerkships had academic performance generally similar to students who had full clerkships, a finding consistent with other published data [4]. There were only two areas of significant difference between the transitional class and the standard class. The FM and RPCT clerkship directors believed the lower end-of-clerkship exam performance was attributable to the shortened duration, but the psychiatry clerkship director attributed the lower number of A’s in the transitional year to a longstanding effort to reduce grade inflation rather than the shortened duration. The overwhelming majority of students (97%) felt that adopting the transitional year was the right approach to provide more time for electives, away rotations, and to prepare and take Step 2 exams before ERAS opened. Likewise, administrators found the M4 scheduling process to be easier with the additional weeks.

Clerkship directors and students felt the additional time in the fourth year allowed for increased confidence in specialty choice, increased confidence in approaching Step 2 CK, and the ability to take Step 2 CK earlier. However, clerkship directors reported uncertainty about whether the benefits of the change were enough to offset their concerns. The fact that clerkship directors experienced the transitional year as more problematic than other stakeholders may reflect that the work required to adjust and manage shortened clerkships largely fell to them, but they experienced no direct benefits. This suggests that some adverse consequences might be mitigated by anticipating the differential impact of a change for those who experience an imbalance between the effort required and the rewards experienced. If such imbalances are identified in advance, efforts to improve this can be included when changes are planned.

Our evaluation has the benefit of using routinely collected information and considering multiple measures and the perspectives of multiple stakeholders. A limitation is that the evaluation includes only one school and only one class of medical students. This cohort was similar to previous and subsequent classes; however, the sample size still remains low. We also only had follow-up surveys from only half of clerkship directors. Finally, this study was limited in its focus on the transitional year impacts only. As the intervention was 1 year, further follow up was not feasible.

Considering all outcomes together, we conclude that shortening clerkships by 1 week was a successful tool for our curriculum change. This strategy may need to be used again as other curriculum needs or circumstances affect clinical training. It is reassuring to have verification that non-inferior educational outcomes can be attained. In addition, the confirmation that a curriculum change has differential impact on stakeholders is important. Anticipating these differential effects can help programs plan more effectively by identifying where adverse impacts from curriculum changes might occur and developing plans to mitigate them. For all curriculum changes there are likely to be some costs to students, faculty, administrators, and the system as a whole and considering them is important to determine if benefits outweigh those costs.

## Data Availability

The data used and/or analyzed during the current study are available from the corresponding author on reasonable request.
